# 2020 Year of the nurse and the midwife: a call for strengthening midwifery in response to South Korea’s ultra-low birth rate

**DOI:** 10.4069/kjwhn.2020.12.03

**Published:** 2020-12-23

**Authors:** Yun Mi Kim

**Affiliations:** College of Nursing, Gachon University, Incheon, Korea

**Keywords:** Birthing centers, Birth rate, Midwifery

## Abstract

Along with the low birth rate in Korea, the aging of mothers is progressing very rapidly. Recent studies have reported that the obstetric infrastructure is crumbling due to the accelerating closures of obstetric medical institutions resulting from the low birth rate and low reimbursement rates for obstetric procedures. The number of birth centers has also decreased, but women’s interest in natural birth has actually increased, such that deliveries at birth centers now account for 11.8% of deliveries in obstetric clinics. In the Netherlands, Japan, and the United Kingdom, initiatives to promote natural birth through care provided by midwives increased the rate of natural births, decreased the number of cesarean sections, and lowered the rate of postpartum complications. In light of these examples, South Korea should also encourage natural delivery by midwives. A national support system for midwife applicants is necessary, and the requirements for institutions that train midwives should be revised. Independent birth centers should have emergency prescription privileges, and women should be given the choice to have a natural delivery by creating birth centers within hospitals.

## Introduction

According to the Presidential Committee on Ageing Society and Population Policy, the budget to address the low birth rate in South Korea has increased by 21.1% per year on average since 2011, reaching 209.5 trillion Korean won (approximately 190.1 billion US dollars) in total during the last 10 years, but the total fertility rate nonetheless decreased by 0.32, from 1.24 in 2011 to 0.92 in 2019. South Korea’s total fertility rate (0.92) was the lowest among 37 Organization for Economic Co-operation and Development (OECD) member countries [1].

In South Korea, the total number of births in 2019 was 300,787, which is 9.2% lower than the corresponding number in 2018 (327,119). The number of obstetric medical institutions also decreased dramatically from 808 in 2010 to 541 in 2019 [2].

Along with the low birth rate, advanced maternal age in South Korea has reached an alarming level, with more than one mothers in five being older than 35 years. The obstetric infrastructure in South Korea is crumbling due to the accelerating closures of obstetric hospitals resulting from the low birth rate and low reimbursement rates for obstetric procedures [3]. In the last 10 years, around 17% of obstetrics and gynecology residents have left their training programs each year, and even obstetricians are giving up on performing deliveries. Thus, the number of delivery rooms is decreasing, and the remaining departments of obstetrics are clustered in the metropolitan areas of Seoul, Gyeonggi Province, and Busan [3]. In response to this situation, the government has designated some rural parts of the country as vulnerable areas since 2011 and provides funds to establish and operate obstetric facilities [4]. However, these measures have not halted the collapse of obstetric infrastructure nor the decrease in the number of obstetric facilities [2,3].

In addition to hospitals and obstetric clinics, the number of birth centers where deliveries occur has steadily dropped from 126 in 2000 to 34 in 2014, 18 in 2016, 16 in 2017 and 2018, and 15 in 2019. There were 652 births in birth centers in 2010; this number increased to 1,260 in 2012 and 1,226 in 2016, before plunging to 912 in 2017, 712 in 2018, and 683 in 2019 [2,5].

In 2019, there were 683 deliveries in 15 birth centers, corresponding to an average of 45.5 deliveries per birth center. Although this value is much lower than the average of 385 deliveries per hospital (100,135 births in 260 hospitals), the focus should be on the fact that 11.8% of deliveries at obstetric clinics occurred in birth centers [2].

According to previous analyses, important factors contributing to women’s interest in delivering in a birth center include the trend for more women to refuse over-treatment that commonly occurs in-hospital settings and desiring baby- and family-focused natural birth that they can be active partners in [6].

This article first discusses natural birth led by midwives in Australia, the Netherlands, Japan, France, and the United Kingdom, with a focus on mothers’ satisfaction and incidence of postpartum complications. This is followed by the second topic, the urgent need to promote midwives in South Korea.

## Natural birth led by midwives in other countries

In a literature review that compiled 23 quantitative studies and nine qualitative studies from around the world about birth outcomes in birth centers, mothers who experienced delivery in birth centers reported trust in the professionalism of midwives, a comfortable and natural birth experience, and satisfaction with personal nursing care from midwives [7].

In Queensland, Australia, delivery by midwives was found to be cost-effective, as the establishment of trust between mothers and midwives reduced women’s fear of delivery, which led to an increased rate of natural delivery and a lower rate of cesarean sections [8]. In the Netherlands, the number of midwives increased more rapidly than the number of obstetricians from 1998 to 2007, and the proportion of births in hospitals attended by midwives increased from 8% to 26% during this period [8]. A Dutch cohort study with 223,739 pregnant women found that women who received midwife-led care had lower rates of severe acute maternal morbidity, postpartum bleeding, and placental detachment than women who received obstetrician-led care. Based on these results, the Dutch cohort study found that there is no evidence that delivery by midwives is riskier if the country has a well-established maternal care system with standards to categorize the level of risk of high-risk pregnancies and a transfer system to higher-level hospitals in emergency situations, such as postpartum bleeding [9,10].

Japan experienced a shortage of obstetricians earlier than South Korea, starting in the 1990s. As a result, Japan experienced the social phenomenon of “birth refugees,” or women who did not have anywhere to deliver due to closure of regional obstetric clinics. This phenomenon resulted in a deterioration of maternal and child health, including suicide among pregnant women, mental health disorders, postpartum depression, and an increased rate of child abuse, which led to the enactment of the Comprehensive Support System for Children and Childrearing based on the Act on Children and Childrearing Support in Japan [11]. The Japanese government recommended using midwives as a measure to resolve the abovementioned issues. This was because midwives can provide continuous care for women from the prenatal to postpartum periods, and are qualified personnel who can protect the health of mothers and babies as medical staff in collaboration with physicians. In addition, an in-hospital birth center system has been actively adopted, through which midwives, together with physicians, can lead the delivery while ensuring respectful care for the family and can provide care up to 1 month postpartum. These birth centers are facilities where midwives, not physicians, help with delivery and are usually utilized by pregnant women who are younger than 35 years or who have a low risk of hypertension or diabetes. Prenatal check-ups and care until delivery are received in obstetric clinics, and women can then choose to deliver either in a birth center or a hospital so that a healthy delivery can be supported by natural birth led by midwives [12,13]. Currently in South Korea, the *Houm* Birthing center and *Houm* OB/GYN Clinic (http://houm.co.kr) exist as an example of a birth center and an obstetrician operating in close collaboration.

France is well known for its efforts to overcome low birth rates through policy measures, which have been implemented in various forms for over 100 years. All medical, hospitalization, and treatment fees that occur after the sixth month of pregnancy are covered by public health insurance (pregnancy insurance) with no out-of-pocket cost. The first prenatal check-up and postpartum check-up have to be done by physicians, but other prenatal and postpartum check-ups can be done by either midwives or physicians [12]. Another example is the United Kingdom. The National Health Service in the United Kingdom has hired 21,000 midwives, compared to 4,710 obstetrics/gynecology (OB-GYN) physicians. Unlike midwives in the United States, midwives in the United Kingdom operate as independent practitioners. They can prescribe drugs needed for delivery and have prescription rights for pregnant women and babies through standard treatment orders, whereby the first prescription is made by a physician, and subsequent ones by midwives [12]. However, the shrinking workforce of midwives following the retirement of midwives currently in their 50s and 60s has put maternal care at risk. The Department of Health and Social Care in the United Kingdom has stated that valuing midwives is vitally important in terms of health care for pregnant women, and is also making efforts to reduce the number of cesarean sections, excluding cases where natural birth is impossible or unsafe [14].

## Training midwives in South Korea: an imperative for maternal-child health in an ultra-low birth rate era

In order for South Korea to reap the benefits of the midwifery systems that have been implemented internationally, it is vitally urgent to implement a sustainable system of training a sufficient number of capable midwives. However, the number of midwives in training is small and this is related in part, to limitations in designated training centers. According to the Korean Midwives Association (KMA), there were seven trainees in Busan Ilsin Hospital, two in Gyeongbuk Andong Sungso Hospital, and four in Seoul Gangbuk Samsung Hospital in 2019; these trainees received training for 1 year, and 12 passed the exam in 2020, excluding one who failed [15]. Busan Ilsin Hospital is the only training institution that admits applicants from outside of their hospital, but the training program will be discontinued after completing education of current trainees in 2020, due to financial difficulties. The KMA and Busan Ilsin Hospital tried to continue the training program with financial subsidy from the government, but did not receive government support. This is in stark contrast to governmental support for nursing assistant training. Applicants for nursing assistant positions can open a vocational competency development account at the Employment Information Service and receive 80% to 100% support for a year of training expenses to become a nursing assistant, depending on their amount of health insurance co-pay. They also receive an additional training allowance if their attendance is higher than 80% [16]. Compared to the government subsidy given to training nursing assistants and considering the important contributions of midwives to maternal-child health through their social competency and scope of practice, the fact that there is no government support to train midwives should be carefully reconsidered, given the need to overcome the ongoing serious problems of an ultra-low birth rate in South Korea.

The need to evaluate the adequacy of medical care in Korea is another issue. Although Korea's Article 38, Clause 1, Attached Table 5 of the Medical Act Enforcement Regulations stipulates that more than one-third of nurses in obstetrics should be midwives, there are no enforcement or intervention measures to support this specification. In 2019, 87% of vaginal births in Korea occurred in OB/GYN specialized hospitals or clinics [17].

Due to issues such as the reimbursement rates for obstetrics being lower than the costs [3], nursing personnel in women’s hospitals (specializing in reproductive health) and OB/GYN clinics are being replaced by nursing assistants. The stark fact of these facilities is that not a single registered nurse is working, except for nurse managerial personnel, not to mention midwives [18]. While other OECD countries such as Australia and the Netherlands train midwives to take charge of normal deliveries [8-10], women in South Korea, also an OECD country, are generally unaware of the serious reality that most of the staff in women’s hospitals where they deliver may be nursing assistants [17,18].

Government support for the midwife training process, which costs approximately 24 million Korean won (nearly 22,000 US dollars) per person per year as of mid-December 2020, can be an important strategy for responding proactively to Korea's low birth rate. Moreover, the requirements for midwife training institutions need to be changed. Current legal requirement that the institution needs to be a training hospital for OB/GYN and pediatrics should be removed, as the number of such training hospitals is rapidly decreasing. The stipulation that the institution must perform more than 100 deliveries per month should also be relaxed, as the number of deliveries is decreasing due to the very low birth rate. As such, the range of institutions should be expanded to hospitals that are certified as medical facilities and perform more than 50 deliveries per month. Another alternative model is possibly adapting the training model of resident physicians, who work and are duly paid for their on-site training (80 hours/week). In similar context, nurses, already a licensed professional, should also be able to work and receive nominal payment as a midwife-in-training. If it were to be possible to employ labor and delivery unit nurses and offer concurrent midwifery training, i.e., aligning the same model that exists for resident training, this would facilitate more midwife training programs to function. The KMA can implement midwife training programs with these hospitals by signing memoranda of understanding. The government should also regulate and financially support obstetric hospitals so that they can abide by the current regulation stipulating that more than one-third of the nursing staff should be midwives.

In Korea, a recent study found that midwives who work in hospitals reported considerably lower professionalism and job satisfaction than those who work in birth centers [19]. In order for midwives working in hospitals to effectively accomplish their main responsibilities as midwives, the in-hospital birth center operation model, where midwives take charge of normal pregnancies, is needed in South Korea. For midwives who operate independent birth centers, the British model where midwives have prescription and admission privileges for emergency situations such as postpartum bleeding, is necessary to ensure safety management for women who may need critical care.

## Conclusion

The rapid crumbling of the obstetric infrastructure due to the ultra-low birth rate is a serious reality that South Korea faces. In order to overcome this situation, the Korean government should support the training of capable midwives and establish a system that gives independent birth centers emergency standard prescription privileges, as in other countries. Women should be given the choice to receive midwife-led care for normal pregnancies by establishing birth centers in hospitals. If a pregnancy becomes high-risk, establishing a system to quickly transfer the patient to a hospital is also needed. These strategic measures will allow midwives to play a pivotal role in counter-acting the ongoing crisis of low birth rate in South Korea.
